# The Role of Congeniality in Multiple Imputation for Doubly Robust Causal Estimation

**DOI:** 10.1002/sim.70363

**Published:** 2026-01-23

**Authors:** Lucy D'Agostino McGowan

**Affiliations:** ^1^ Department of Statistical Sciences Wake Forest University Winston‐Salem North Carolina USA

**Keywords:** causal inference, doubly robust, inverse probability weighting, missing data, multiple imputation

## Abstract

This paper provides clear and practical guidance on the specification of imputation models when multiple imputation is used in conjunction with doubly robust estimation methods for causal inference. Through theoretical arguments and targeted simulations, we demonstrate that if a confounder has missing data, the corresponding imputation model must include all variables appearing in either the propensity score model or the outcome model, in addition to both the exposure and outcome, and that these variables must appear in the same functional form as in the final analysis. Violating these conditions can lead to biased treatment effect estimates, even when both components of the doubly robust estimator are correctly specified. We present a mathematical framework for doubly robust estimation combined with multiple imputation, establish the theoretical requirements for proper imputation in this setting, and demonstrate the consequences of misspecification through simulation. Based on these findings, we offer concrete recommendations to ensure valid inference when using multiple imputation with doubly robust methods in applied causal analyses.

## 1 Introduction

In observational studies attempting to estimate causal effects researchers increasingly rely on methods that combine multiple analytical models to estimate treatment effects. One common approach uses propensity score models, which estimate the probability of treatment assignment given observed covariates to help balance treatment and control groups. Alternatively, researchers can fit outcome models that directly relate observed covariates to the outcome distribution, relying on the model specification to control for confounding. Doubly robust estimators, which combine both approaches, have become increasingly popular as they offer protection against model misspecification by combining propensity score models with outcome regression models [1, 2, 3, 4, 5]. Targeted maximum likelihood estimation (TMLE) has emerged as another doubly robust approach that combines the propensity score and the outcome modeling while offering additional efficiency properties [6]. Applied researchers frequently encounter missing data in covariates or outcomes. While multiple imputation (MI) has emerged as a common solution for handling such missingness under the missing at random (MAR) assumption, there remains limited guidance on how to properly specify imputation models when working with these combined analysis approaches.

For handling propensity score methods with multiply imputed data, the preferred strategy involves generating m imputed datasets, estimating the propensity score and treatment effect in each one, and then combining the m resulting average treatment effect estimates using Rubin's rules, an approach sometimes termed the “within” approach in the literature. This approach has been shown to outperform alternatives that, e.g., pool propensity scores across imputations before estimating treatment effects [7, 8]. However, while the general structure of this approach is now well accepted, the accompanying studies offer limited and inconsistent guidance on the specification of the imputation model itself, particularly when multiple analysis models are involved, as is the case with doubly robust estimators.

The imputation model's specification becomes especially critical when working with doubly robust estimators. These estimators incorporate both a propensity score model and an outcome model, and are consistent if either model is correctly specified [2, 3]. This dual‐model structure creates unique challenges for imputation model specification. Existing methodological literature provides fragmented guidance across different statistical frameworks, making systematic implementation difficult for practitioners. In theory, the imputation model must preserve the joint distribution of all variables that will be used in any subsequent analysis models to ensure congeniality [9, 10]. Congeniality refers to the compatibility between the imputation model and the subsequent analysis models, specifically, whether the imputation model adequately captures the relationships among variables as they are included in the analysis models. Failure to maintain this congeniality can introduce bias even when the analysis models themselves are correctly specified.

A common oversight in applied research is the exclusion of important variables or their functional forms from imputation models. For example, the simulation study by Mitra and Reiter [11], which aimed to compare MI–propensity score integration strategies, excluded the outcome variable from the imputation model entirely. As noted by Penning de Vries and Groenwold [12], this decision invalidates the results and conclusions of that comparison, because excluding the outcome from the imputation model leads to biased estimates [13]. Similarly, [14] examine multiple imputation under several modeling strategies, but incorrectly excluded the exposure variable from their imputation model (which included only confounders and the outcome). The authors attributed their suboptimal MI results to poor model fit, while the likely underlying issue stemmed from a lack of congeniality between their imputation and analysis models. This pattern of observing bias when comparing results to MI methods without explicitly connecting it to congeniality principles appears repeatedly in the literature. In another recent example, Dashti et al. [15] compared various MI scenarios paired with TMLE and identified several scenarios where default MI methods yielded biased results, while methods that matched the complexity of the analysis models performed better. While their observations were likely fundamentally driven by compatibility between imputation and analysis models, the connection to the broader principle of congeniality and its systematic application across different doubly robust frameworks was not made explicit. Collectively, these examples illustrate the need for systematic guidance that makes the connection between theoretical congeniality principles and practical implementation explicit. While we highlight these specific studies, it is important to note that they are commendable for providing sufficient methodological detail to allow readers to evaluate their imputation models, a level of transparency that is, unfortunately, rare in clinical research, suggesting that such misspecification issues are likely far more widespread than the published literature would indicate. When estimating causal effects, intuition might suggest that certain variables could be safely omitted from imputation models, such as precision variables that affect only the outcome. However, when a confounder has missing values, these variables must be included in the corresponding imputation models when they appear in subsequent analyses, and their inclusion must match the functional form in the subsequent models.

This paper provides theoretical and empirical guidance on what must be included in the imputation model when MI is combined with doubly robust estimation methods, focusing on settings with binary treatment and continuous pre‐exposure covariates and outcome. We show through theoretical argument and simulations that when using MI in conjunction with propensity score estimation and outcome modeling, the imputation model must include all variables used in either the propensity score model or the outcome model(s), and these variables must appear in the same functional form as they do in the subsequent analyses [16, 17, 18]. We demonstrate that violating these conditions leads to biased estimates of treatment effects, even when both components of the doubly robust estimator are correctly specified. Our contribution consolidates theoretical requirements into practical implementation guidelines while quantifying the empirical consequences of specification violations.

We organize the remainder of this paper as follows. First, we present the mathematical framework for doubly robust estimation and multiple imputation. Next, we develop the theoretical requirements for proper imputation model specification in this context, with particular attention to the challenges posed by combining multiple analysis models. We then use targeted simulations to illustrate how violations of these principles affect bias and efficiency. Finally, we offer practical recommendations for analysts using MI alongside doubly robust estimators in applied causal inference settings.

## 2 Mathematical Framework

We consider a standard potential outcomes framework for causal inference. Let X∈{0,1} denote a binary exposure, Y∈ℝ an outcome, and $Z\in {\mathbb{R}}^{p}$ a vector of pre‐exposure covariates. For each individual, let Y(1) and Y(0) denote the potential outcomes under treatment and control, respectively. Our estimand is the average treatment effect (ATE),

Δ=𝔼[Y(1)−Y(0)].

**FIGURE 1:**
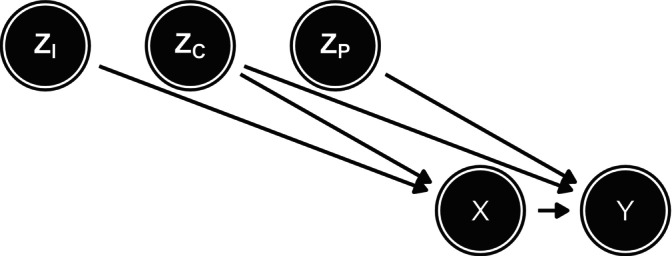
Directed acyclic graph describing the relationship between X, Y, and Z where $Z_{I}$ represent instruments, $Z_{C}$ confounders, and $Z_{P}$ precision variables.

To clarify the causal roles of covariates, we partition the covariate vector Z into three disjoint subsets:

$$Z=(Z_{I},\;Z_{P},\;Z_{C}),$$

where $Z_{I}$ denotes instruments (variables that causally affect X but not Y), $Z_{P}$ denotes precision variables (variables that causally affect Y but not X), and $Z_{C}$ denotes confounders (variables that causally affect both X and Y). Figure 1 is a directed acyclic graph (DAG) that displays these relationships.

To identify Δ, we rely on the following standard assumptions:

1. Consistency: Y=Y(X), that is, the observed outcome corresponds to the potential outcome under the observed exposure,
2. Exchangeability (Unconfoundedness): $Y(1),Y(0)⊥\;\;\;⊥X|Z_{C}$, and
3. Positivity: $0<\mathbb{P}(X=1|Z_{C})<1$.

Under these assumptions, the ATE can be identified using several estimation strategies. One common approach is inverse probability weighting (IPW), where we define the propensity score as

$$\pi (Z_{C})=\mathbb{P}(X=1|Z_{C}),$$

and construct the IPW estimator as

(1) $${\hat{\Delta }}_{\text{IPW}}=\frac{1}{n}\overset{n}{\underset{i=1}{\sum }}\left[\frac{X_{i}Y_{i}}{\pi (Z_{C,i})}-\frac{(1-X_{i})Y_{i}}{1-\pi (Z_{C,i})}\right].$$

We can optionally fit a doubly robust estimator, which combines the IPW and outcome regression approaches. Often this involves fitting separate outcome regression models for the exposed and unexposed groups, then combining these results with inverse probability weights. While there are several approaches to incorporating both propensity scores and outcome regressions, in this paper, we focus on augmented inverse probability weighting [3, 4, 19]. This doubly robust estimator is defined as follows:

(2) $$\begin{array}{cc} {\hat{\Delta }}_{\text{DR}} & =\frac{1}{n}\overset{n}{\underset{i=1}{\sum }}\left[\mu_{1}(Z_{C,i})-\mu_{0}(Z_{C,i})\right]\\ & \;+\frac{1}{n}\overset{n}{\underset{i=1}{\sum }}\left[\frac{X_{i}\left\{Y_{i}-\mu_{1}(Z_{C,i})\right\}}{\pi (Z_{C,i})}-\frac{(1-X_{i})\left\{Y_{i}-\mu_{0}(Z_{C,i})\right\}}{1-\pi (Z_{C,i})}\right], \end{array}$$

where $\mu_{1}(Z_{C})$ and $\mu_{0}(Z_{C})$ are predicted outcomes of a regression model fit among the exposed (X=1) and unexposed (X=0) respectively. Note if all the same variables are included in both outcome models, fitting these models separately among the exposed and unexposed effectively includes an interaction term between all variables and the exposure.

An advantage of doubly robust estimators is that they remain consistent if either the propensity score model (π) or the outcome models (μ) are correctly specified. Even when both models use the same covariates $Z_{C}$, this property may be useful in that it ensures consistency as long as at least one model correctly captures the functional relationship between variables. It is also possible to estimate μ using both $Z_{C}$ and $Z_{P}$, as including variables that are predictive of the outcome (but not the exposure) can improve the efficiency of the estimator without introducing bias [3, 20, 21, 22, 23, 24]. In fact, Craycroft et al. [25] even recommend including both confounders ($Z_{C}$) and precision variables ($Z_{P}$) when estimating the propensity score model π. They also demonstrate that including instruments ($Z_{I}$) in the propensity score model is not recommended.

## 3 Missing Data and Multiple Imputation

Data may be missing in the outcome Y and/or covariates Z. Let $M^{Y}$ and $M^{Z}$ denote missingness indicators, where $M^{V}=1$ if variable V∈{Y,Z} is missing. Let $O=(Y^{*},Z^{*})$ represent the observed data, where for any variable V, $V^{*}=V$ if $M^{V}=0$, and is missing otherwise.

We assume that the data are MAR, meaning that the probability of missingness depends only on the observed data. Specifically:

$$\mathbb{P}(M^{V}=1|X,Y,Z)=\mathbb{P}(M^{V}=1|O).$$

Under this MAR assumption, MI is a valid strategy for handling missing data. MI proceeds by specifying a set of imputation models. These models are used to generate m completed datasets by drawing from the posterior predictive distribution of the missing values. For a variable V with missingness, the imputation model aims to draw from its posterior predictive distribution:

$$\mathbb{P}(V_{mis}|O,\theta_{V})$$

where $\theta_{V}$ represents the parameters of the imputation model for variable V. For example, when imputing a continuous confounder $Z_{C}$ with missing values, a linear model might be specified as:

(3) $$Z_{C}=\beta_{0}+\beta_{1}X+\beta_{2}Z_{I}+\beta_{3}Z_{p}+\beta_{4}Y+\varepsilon ,\;\varepsilon ∼𝒩(0,1),$$

where draws from this model are used to generate stochastic imputations in each multiply imputed dataset. Equation (3) assumes $Z_{I}$ and $Z_{P}$ both appear in the subsequent analysis models (i.e., the propensity score and/or outcome models) in linear form. Of course, in practice the imputation model can include nonlinear terms and/or additional interactions (and in fact it must if those relationships exist in the subsequent analysis models). In addition to parametric models like the linear regression represented here, other imputation approaches can be employed, including non‐parametric methods such as predictive mean matching, classification and regression trees (CART), random forests, or k‐nearest neighbors [26].

When fitting imputation models when using doubly robust methods with a binary exposure, we recommend fitting the model separately within each exposure group to ensure that the imputation model is congenial with the outcome model that is typically fit separately by exposure status (or with an interaction). For example, when imputing the continuous confounder $Z_{C}$ separately within each exposure group X=1 and X=0, we would specify two distinct models:

$$\begin{array}{ll} \text{For}\;X=1:\;Z_{C} & =\beta_{0}^{\left(1\right)}+\beta_{1}^{\left(1\right)}Z_{I}\\ & \;+\beta_{2}^{\left(1\right)}Z_{P}+\beta_{3}^{\left(1\right)}Y+\varepsilon^{\left(1\right)},\;\varepsilon^{\left(1\right)}∼𝒩(0,1),\\ \text{For}\;X=0:\;Z_{C} & =\beta_{0}^{\left(0\right)}+\beta_{1}^{\left(0\right)}Z_{I}\\ & \;+\beta_{2}^{\left(0\right)}Z_{P}+\beta_{3}^{\left(0\right)}Y+\varepsilon^{\left(0\right)},\;\varepsilon^{\left(0\right)}∼𝒩(0,1). \end{array}$$

Each set of coefficients $\beta^{\left(x\right)}$ is estimated using only data from units with exposure status X=x. This stratified approach ensures that the imputation model remains congenial with outcome or propensity score models that are fit separately by exposure group or that include interactions with X.

These imputation models are used to generate m completed datasets by drawing from the posterior predictive distribution of the missing values. Within each imputed dataset, causal estimands can be computed using IPW alone or the doubly robust methods described above (Equations 1 and 2). Estimates are then combined using Rubin's rules [27]. The pooled point estimate is:

(4) $$\bar{\Delta }=\frac{1}{m}\overset{m}{\underset{j=1}{\sum }}{\hat{\Delta }}^{\left(j\right)},$$

with total variance:

$$T=Ū+\left(1+\frac{1}{m}\right)B,$$

where $Ū=\frac{1}{m}\sum_{j=1}^{m}U^{\left(j\right)}$ is the average within‐imputation variance, and $B=\frac{1}{m-1}\sum_{j=1}^{m}{\left({\hat{\Delta }}^{\left(j\right)}-\bar{\Delta }\right)}^{2}$ is the between‐imputation variance.

## 4 Theoretical Considerations

When implementing multiple imputation, ensuring compatibility between the imputation and analysis models is essential for obtaining unbiased estimates. In causal inference, one might assume that accurately modeling the relationships among the exposure, outcome, and confounders is sufficient, since these are the only variables required to identify the causal effect. However, this assumption can break down when stochastic imputation (like MI) is used. Variables that are marginally independent in the data‐generating process can become conditionally dependent once we condition on their common effect. If such variables are omitted from the imputation model, it can lead to biased estimates, even when the analysis models are correctly specified. We need to carefully consider the full joint distribution of variables in the analysis, not just those required for identification.

Consider our data‐generating mechanism (Figure 1) where X causes Y, $Z_{C}$ are confounders affecting both X and Y and $Z_{P}$ are precision variables that only affects Y. While $Z_{C}$ and $Z_{P}$ are marginally independent, they will become conditionally associated if conditioned on Y because they are both causes of Y.

To motivate our recommendations for specifying imputation models in causal inference with missing data, we consider two scenarios:

i. missing confounders $Z_{C}$ and
ii. missing outcome Y.

In all cases, we assume the missingness mechanism satisfies the MAR assumption. We estimate the propensity score model as $\pi (Z_{C})$. The outcome component of the doubly robust estimator is estimated as $\mu_{0}(Z_{C},Z_{P})$ and $\mu_{1}(Z_{C},Z_{P})$ as in Equation (2), and combined across imputed datasets as described in Equation (4).

### 4.1 Imputing Missing Confounders

Suppose we have missing values in one of the confounders, denoted $Z_{C(j)}$ indicating that the jth column of $Z_{C}$ (the matrix of all confounders) has missing entries. When handling missing values in a confounder such as $Z_{C(j)}$, the imputation model must include all variables present in the analysis models to avoid bias. This fundamental principle extends beyond merely including obvious causal components (X and Y); it necessitates including all auxiliary variables, such as precision variables $Z_{P}$, that appear in the subsequent models. The rationale stems from the need to preserve the correct correlation structure between the imputed and observed variables. When imputing $Z_{C(j)}$, including Y in the imputation model is essential for unbiased estimation [13]. However, this inclusion induces a conditional association between variables that may be marginally independent in the data‐generating process (e.g., between $Z_{C(j)}$ and $Z_{P}$, see Figure 1).

Doubly robust estimators provide theoretical protection against model misspecification, requiring only one of two models, either the propensity score model or the outcome model, to be correctly specified for consistent estimation. However, this double robustness property does not extend to protecting against imputation misspecification, even for variables that appear exclusively in the outcome model and not in the propensity score model.

Examining Equation (2) reveals that it comprises two components: The estimated causal effect derived from the outcome model (first term) and the weighted residuals from the outcome models (the weighted difference between observed outcomes and their model‐predicted values, the second term). When precision variables ($Z_{P}$) are omitted from the imputation model but included in the outcome model, the propensity score model itself remains correctly specified and unaffected by $Z_{P}$. However, within strata defined by exposure and confounders, the residuals from the outcome model will average to zero (a fundamental property of least squares estimation). This mathematical inevitability nullifies the second term of Equation (2), effectively preventing the correctly specified propensity score model from contributing to the estimate at all. The estimation then relies solely on the outcome model's contribution, which yields biased estimates because it uses incorrectly imputed data.

The proper imputation model for $Z_{C(j)}$ must therefore reflect its conditional distribution given all variables in subsequent analysis models: The exposure (X), the outcome (Y), other confounders ($Z_{C(j)}$), and precision variables ($Z_{P}$).

Since the doubly robust estimator in Equation (2) involves fitting separate outcome models for exposed and unexposed groups, we can separately fit the imputation model among the exposed and unexposed to account for this.

### 4.2 Imputing Missing Outcomes

As above, when imputing Y it is intuitive that X is necessary in the imputation model. Likewise, if an estimator such as Equation (2) is used that has two outcome models depending on exposure status, the imputation models should also be fit separately among exposure groups. Interestingly, when Y is the variable with missing values requiring imputation, excluding $Z_{P}$ from the imputation model does not introduce bias in estimating the X‐Y relationship, even when $Z_{P}$ is included in the analysis model as long as separate imputation models are appropriately fit by exposure status. Likewise, excluding $Z_{C}$ also will not induce bias as long as imputation models are fit separately by exposure status.

This contrast highlights an important asymmetry in how misspecified imputation models affect causal estimates. When imputing confounders, failing to account for conditional associations induced by the collider structures lead to bias. However, when imputing outcomes, omitting variables that are in the outcome model (like confounders or precision variables) may affect efficiency, but will not bias the causal parameter of interest when using a doubly robust estimator, as long as the imputation model is properly fit within exposure strata.

In the following section, we empirically demonstrate these theoretical considerations. In addition to demonstrating the bias introduced by leaving necessary variables out of the imputation model, we also examine the impact of including the right variables, but with the incorrect functional form.

## 5 Simulation

We conducted a simulation study to demonstrate the impact of (1) leaving variables out of the imputation model, and (2) including the right variables but with an incorrect functional form. For each scenario, we examined four different sample sizes (100, 500, 1000, and 2000) and ran 500 simulations for each. This number was chosen to ensure adequate precision in our simulation estimates, even for the smallest sample size.

### 5.1 Primary Scenarios

**FIGURE 2:**
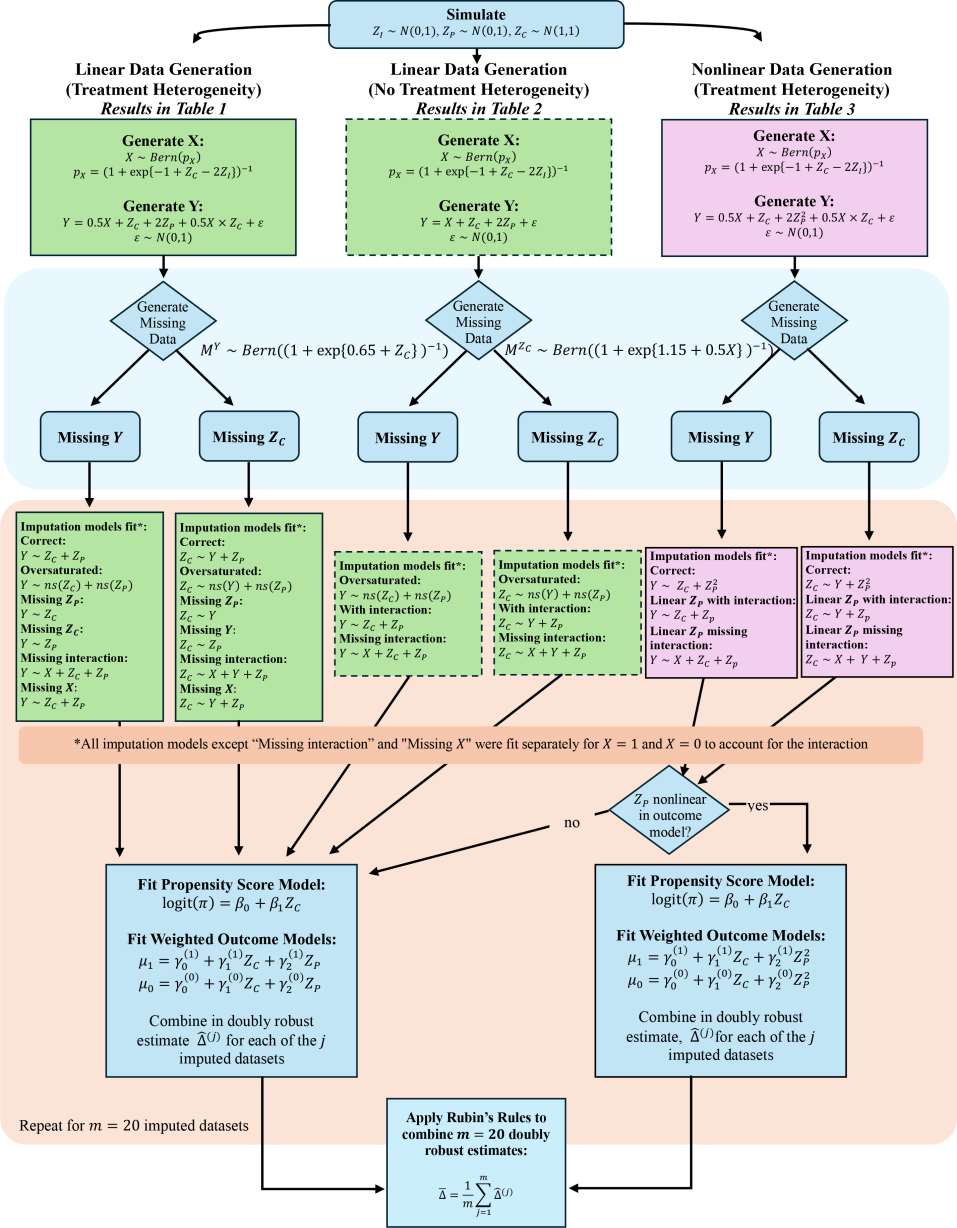
Simulation schema.

Figure 2 presents the overall simulation schema. For each scenario, we first generated three normally distributed variables $Z_{I}∼N(0,1)$, $Z_{P}∼N(0,1)$, and $Z_{C}∼N(1,1)$. The binary treatment variable X was generated from a Bernoulli distribution with probability:

$$p_{X}={\left(1+\exp\{Z_{C}-2Z_{I}\}\right)}^{-1}.$$

The outcome Y was generated according to:

$$\begin{array}{ll} & \text{Linear case with treatment heterogeneity:}\\ & \;Y=0.5X+Z_{C}+2Z_{P}+0.5X\times Z_{C}+\varepsilon \\ & \text{Linear case without treatment heterogeneity:}\\ & \;Y=X+Z_{C}+2Z_{P}+\varepsilon \\ & \text{Nonlinear case with treatment heterogeneity:}\\ & \;Y=0.5X+Z_{C}+2Z_{P}^{2}+0.5X\times Z_{C}+\varepsilon , \end{array}$$

where ε∼N(0,1). To simulate missing data mechanisms, we introduced missingness for Y and $Z_{C}$ with the following probabilities:

$$\begin{array}{ll} \mathbb{P}(M^{Y}=1) & ={\left(1+\exp\{0.65+Z_{C}\}\right)}^{-1},\\ \mathbb{P}(M^{Z_{C}}=1) & ={\left(1+\exp\{1.15+0.5X\}\right)}^{-1}, \end{array}$$

where $M^{V}=1$ indicates that variable V is missing. The above values were chosen to create approximately 20% missingness in each simulation.

Our propensity score model is specified as:

$$\begin{array}{l} \text{logit}(\pi )=\beta_{0}+\beta_{1}Z_{C}. \end{array}$$

Outcome models are specified as:

$$\begin{array}{ll} \text{Linear case:}\; & \mu_{1}=\gamma_{0}^{\left(1\right)}+\gamma_{1}^{\left(1\right)}Z_{C}+\gamma_{2}^{\left(1\right)}Z_{P},\\ & \mu_{0}=\gamma_{0}^{\left(0\right)}+\gamma_{1}^{\left(0\right)}Z_{C}+\gamma_{2}^{\left(0\right)}Z_{P},\\ \text{Nonlinear case:}\; & \mu_{1}=\gamma_{0}^{\left(1\right)}+\gamma_{1}^{\left(1\right)}Z_{C}+\gamma_{2}^{\left(1\right)}Z_{P}^{2},\\ & \mu_{0}=\gamma_{0}^{\left(0\right)}+\gamma_{1}^{\left(0\right)}Z_{C}+\gamma_{2}^{\left(0\right)}Z_{P}^{2}. \end{array}$$

For each missing data pattern, we determined and fitted the imputation models as described in Figure 2 m=20 times using the mice package in R [28]. The “oversaturated” imputation model was split by exposure and included $Z_{P}$ and $Z_{C}$, both fit with natural splines with 3 degrees of freedom, when the outcome was missing and the outcome and $Z_{P}$ fit with a natural spline with three degrees of freedom when the confounder was missing. We then calculated doubly robust estimates ${\hat{\Delta }}^{\left(j\right)}$ for each imputed dataset. These estimates were combined using Rubin's rules to obtain the final estimate as described in Section 2. All data were generated such that the true average treatment effect Δ=1.

### 5.2 Multiple Confounder Scenarios

**FIGURE 3:**
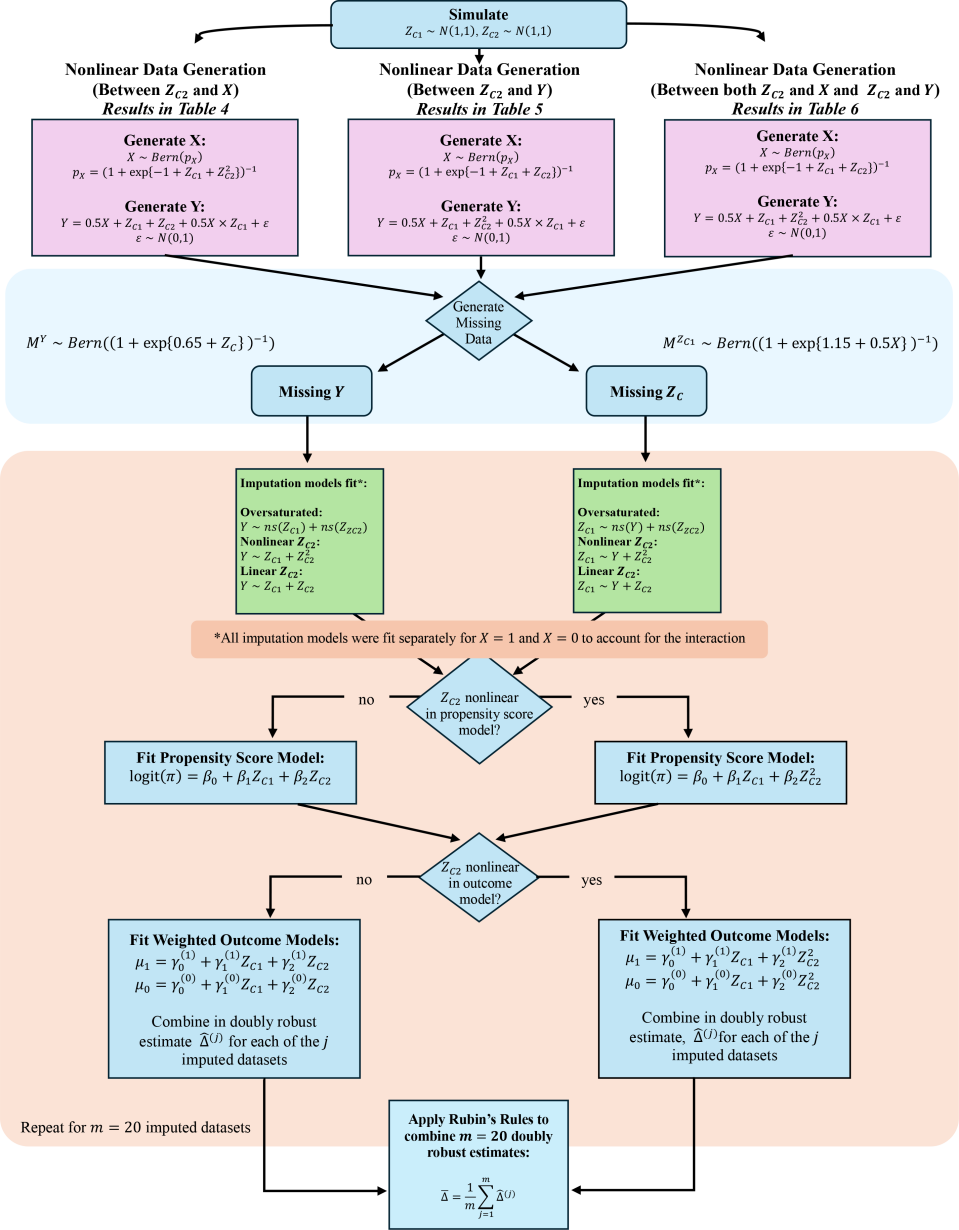
Simulation schema (multiple confounders).

Figure 3 presents the schema for our second simulation study, which extends the framework to include two confounders with the non‐missing confounder having varying nonlinear relationships with the exposure and/or outcome.

For each scenario, we first generated two normally distributed variables: $Z_{C1}∼N(1,1)$ and $Z_{C2}∼N(1,1)$. We examined three data generation scenarios that varied the nonlinear relationships between $Z_{C2}$ and the exposure and/or outcome.

#### 5.2.1 Scenario 1: Nonlinear $Z_{C2}-X$ Relationship

The binary treatment variable X was generated from a Bernoulli distribution with probability:

(5) $$p_{X}={\left(1+\exp\{Z_{C1}+Z_{C2}^{2}\}\right)}^{-1}.$$

The outcome Y was generated according to:

(6) $$Y=0.5X+Z_{C1}+Z_{C2}+0.5X\times Z_{C1}+\varepsilon .$$

#### 5.2.2 Scenario 2: Nonlinear $Z_{C2}-Y$ Relationship

The binary treatment variable X was generated from a Bernoulli distribution with probability:

(7) $$p_{X}={\left(1+\exp\{Z_{C1}+Z_{C2}\}\right)}^{-1}.$$

The outcome Y was generated according to:

(8) $$Y=0.5X+Z_{C1}+Z_{C2}^{2}+0.5X\times Z_{C1}+\varepsilon .$$

#### 5.2.3 Scenario 3: Nonlinear $Z_{C2}-X$ and $Z_{C2}-Y$ Relationships

The binary treatment variable X was generated from a Bernoulli distribution with probability:

(9) $$p_{X}={\left(1+\exp\{Z_{C1}+Z_{C2}^{2}\}\right)}^{-1}.$$

The outcome Y was generated according to:

(10) $$Y=0.5X+Z_{C1}+Z_{C2}^{2}+0.5X\times Z_{C1}+\varepsilon ,$$

where ε∼N(0,1) in all cases.

To simulate missing data mechanisms, we introduced missingness for Y and $Z_{C1}$ using the same probabilities as in Section 4.1:

(11) $$\begin{array}{ll} \mathbb{P}(M^{Y}=1) & ={\left(1+\exp\{0.65+Z_{C1}\}\right)}^{-1}, \end{array}$$

(12) $$\begin{array}{ll} \mathbb{P}(M^{Z_{C1}}=1) & ={\left(1+\exp\{1.15+0.5X\}\right)}^{-1}, \end{array}$$

where $M^{V}=1$ indicates that variable V is missing. Again, the above values were chosen to create approximately 20% missingness in each simulation.

Our propensity score models were correctly specified for each scenario such that Scenarios 1 and 3 included the quadratic $Z_{C2}$ term and Scenario 2 included a linear $Z_{C2}$ term.

Outcome models were similarly correctly specified, such that Scenarios 2 and 3 included the quadratic $Z_{C2}$ term and Scenario 1 included a linear $Z_{C2}$ term.

For each missing data pattern and scenario, we fit three different imputation strategies, with separate models by treatment group X to account for treatment heterogeneity:

1. an “oversaturated” imputation model was split by exposure and included $Z_{C1}$ and $Z_{C2}$, both fit with natural splines with 3 degrees of freedom, when the outcome was missing and the outcome and $Z_{C2}$ fit with a natural spline with three degrees of freedom when $Z_{C1}$ was missing,
2. an imputation model with $Z_{C2}$ included linearly and otherwise correctly specified, and
3. an imputation model with $Z_{C2}^{2}$included and otherwise correctly specified. Each imputation was performed m=20 times using the mice package in R, and doubly robust estimates were combined using Rubin's rules to obtain the final estimate as described in Section 2. All data were generated such that the true average treatment effect Δ=1.

### 5.3 Results

**FIGURE 4:**
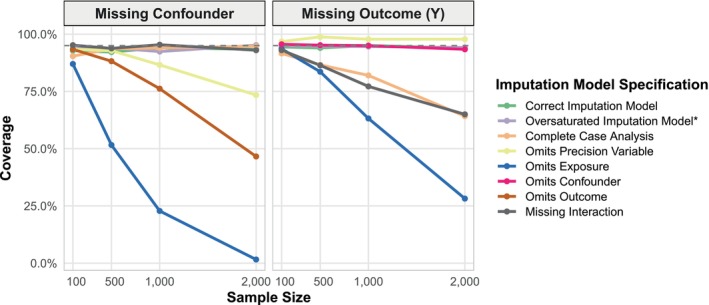
Coverage probability by sample size for doubly robust estimates under different imputation specifications. Data were simulated under the scenario described as “Linear Data Generation (Treatment Heterogenity)” in Figure 2. The dashed line indicates nominal 95% coverage.

We conducted 500 simulation replications for each condition and computed the mean and standard deviation of the estimated treatment effects. Table 1 and Figure 4 show the correctly specified models as well as the impact of omitting X, Y, $Z_{C}$, and $Z_{P}$ from the imputation models. Table 2 shows the impact of the true data‐generating mechanism having homogeneous treatment effects (instead of heterogeneous, as was the default). Table 3 shows the impact of incorrectly specifying nonlinear terms that were included in the analysis model in the imputation model.

**TABLE 1:** Simulation results under heterogeneous treatment effect data generation for the linear data generating mechanism by imputation model specification. The true average treatment effect is 1.

Model specification	Est.	MC SE	Avg. SE	Bias	RMSE	95% Cov.
Missing confounder $(Z_{C})-n=100$						
Correct imputation model	1.01	0.24	0.22	0.01	0.24	0.93
Oversaturated imputation model^a^	0.97	0.25	0.23	−0.03	0.25	0.94
Complete case analysis	0.98	0.26	0.22	−0.02	0.26	0.90
Omits precision variable	0.91	0.26	0.26	−0.09	0.28	0.93
Omits exposure	0.79	0.20	0.23	−0.21	0.30	0.87
Omits outcome	0.85	0.27	0.29	−0.15	0.30	0.93
Missing interaction	1.02	0.22	0.22	0.02	0.22	0.95
Missing Confounder $(Z_{C})-n=500$						
Correct imputation model	0.99	0.11	0.10	−0.01	0.11	0.92
Oversaturated imputation model^a^	0.99	0.10	0.10	−0.01	0.10	0.94
Complete case analysis	0.99	0.11	0.10	−0.01	0.11	0.94
Omits precision variable	0.92	0.11	0.12	−0.08	0.13	0.93
Omits exposure	0.80	0.09	0.10	−0.20	0.22	0.52
Omits outcome	0.87	0.11	0.13	−0.13	0.17	0.88
Missing interaction	0.99	0.11	0.10	−0.01	0.11	0.94
Missing confounder $(Z_{C})-n=1000$						
Correct imputation model	1.00	0.07	0.07	0.00	0.07	0.94
Oversaturated imputation model^a^	1.00	0.08	0.07	0.00	0.08	0.92
Complete case analysis	0.98	0.07	0.07	−0.02	0.08	0.94
Omits precision variable	0.92	0.08	0.09	−0.08	0.11	0.87
Omits exposure	0.80	0.06	0.07	−0.20	0.21	0.23
Omits outcome	0.87	0.08	0.09	−0.13	0.16	0.76
Missing interaction	1.00	0.07	0.07	0.00	0.07	0.95
Missing confounder $(Z_{C})-n=2000$						
Correct imputation model	0.99	0.05	0.05	−0.01	0.05	0.93
Oversaturated imputation model^a^	1.00	0.05	0.05	0.00	0.05	0.95
Complete case analysis	0.99	0.05	0.05	−0.01	0.05	0.95
Omits precision variable	0.92	0.05	0.06	−0.08	0.10	0.73
Omits exposure	0.80	0.05	0.05	−0.20	0.21	0.02
Omits outcome	0.86	0.05	0.07	−0.14	0.15	0.47
Missing interaction	1.00	0.05	0.05	0.00	0.05	0.93
Missing outcome (Y)−n=100						
Correct imputation model	1.02	0.24	0.24	0.02	0.24	0.94
Oversaturated imputation model^a^	0.99	0.27	0.29	−0.01	0.27	0.95
Complete case analysis	1.09	0.25	0.22	0.09	0.26	0.92
Omits precision variable	0.99	0.36	0.42	−0.01	0.36	0.97
Omits exposure	0.84	0.20	0.24	−0.16	0.26	0.94
Omits confounder	0.96	0.28	0.31	−0.04	0.28	0.96
Missing interaction	1.07	0.24	0.24	0.07	0.25	0.93
Missing outcome (Y)−n=500						
Correct imputation model	1.00	0.11	0.11	0.00	0.11	0.94
Oversaturated Imputation Model^a^	0.99	0.11	0.11	−0.01	0.11	0.95
Complete case analysis	1.08	0.11	0.10	0.08	0.13	0.87
Omits precision variable	1.00	0.15	0.19	0.00	0.15	0.99
Omits exposure	0.87	0.09	0.11	−0.13	0.16	0.84
Omits confounder	0.96	0.13	0.14	−0.04	0.13	0.95
Missing interaction	1.08	0.11	0.11	0.08	0.14	0.86
Missing outcome (Y)−n=1000						
Correct imputation model	0.99	0.08	0.08	−0.01	0.08	0.95
Oversaturated imputation model^a^	1.00	0.08	0.08	0.00	0.08	0.95
Complete Case analysis	1.08	0.07	0.07	0.08	0.11	0.82
Omits precision variable	1.00	0.11	0.13	0.00	0.11	0.98
Omits exposure	0.87	0.06	0.08	−0.13	0.15	0.63
Omits confounder	0.96	0.09	0.10	−0.04	0.10	0.95
Missing interaction	1.09	0.07	0.08	0.09	0.11	0.77
Missing outcome (Y)−n=2000						
Correct Imputation Model	1.00	0.05	0.05	0.00	0.05	0.94
Oversaturated imputation model^a^	1.00	0.06	0.05	0.00	0.06	0.94
Complete case analysis	1.08	0.05	0.05	0.08	0.10	0.64
Omits precision variable	1.00	0.08	0.09	0.00	0.08	0.98
Omits exposure	0.87	0.04	0.06	−0.13	0.14	0.28
Omits confounder	0.96	0.06	0.07	−0.04	0.08	0.93
Missing interaction	1.08	0.05	0.05	0.08	0.10	0.65

*Abbreviations: n* = Sample size; Est. = Estimate; MC SE = Monte Carlo Standard Error; Avg. SE = Average Estimated Standard Error; 95% Cov. = 95% Confidence Interval Coverage.

^a^ Oversaturated Imputation Models fit split by exposure, include the outcome, and include the precision and confounder fit with natural splines with three degrees of freedom each.

**TABLE 2:** Simulation results under homogenous treatment effect data generation by the imputation model specification. The true average treatment effect is 1.

Model specification	Est.	MC SE	Avg. SE	Bias	RMSE	95% Cov.
Missing confounder $(Z_{C})-n=100$						
Correct imputation model	1.01	0.23	0.22	0.01	0.23	0.93
Oversaturated Imputation Model^a^	0.96	0.24	0.23	−0.04	0.24	0.94
Complete case analysis	0.99	0.25	0.22	−0.01	0.25	0.91
Missing Interaction	1.02	0.22	0.22	0.02	0.22	0.95
Missing confounder $(Z_{C})-n=500$						
Correct imputation model	1.00	0.10	0.10	0.00	0.10	0.94
Oversaturated imputation model^a^	0.99	0.10	0.10	−0.01	0.10	0.95
Complete case analysis	0.99	0.11	0.10	−0.01	0.11	0.94
Missing Interaction	0.99	0.10	0.10	−0.01	0.10	0.94
Missing confounder $(Z_{C})-n=1000$						
Correct imputation model	1.00	0.07	0.07	0.00	0.07	0.94
Oversaturated imputation model^a^	1.00	0.07	0.07	0.00	0.07	0.94
Complete case analysis	0.99	0.07	0.07	−0.01	0.07	0.95
Missing Interaction	1.00	0.07	0.07	0.00	0.07	0.97
Missing confounder $(Z_{C})-n=2000$						
Correct imputation model	0.99	0.05	0.05	−0.01	0.05	0.94
Oversaturated imputation model^a^	1.00	0.05	0.05	0.00	0.05	0.95
Complete case analysis	1.00	0.05	0.05	0.00	0.05	0.96
Missing Interaction	1.00	0.05	0.05	0.00	0.05	0.95
Missing Outcome (Y)−n=100						
Correct imputation model	1.01	0.24	0.24	0.01	0.24	0.95
Oversaturated imputation model^a^	0.99	0.26	0.29	−0.01	0.26	0.97
Complete case analysis	1.00	0.24	0.22	0.00	0.24	0.94
Missing interaction	0.99	0.24	0.23	−0.01	0.24	0.95
Missing Outcome (Y)−n=500						
Correct imputation model	1.00	0.11	0.11	0.00	0.11	0.95
Oversaturated imputation model^a^	0.99	0.10	0.11	−0.01	0.10	0.96
Complete case analysis	0.99	0.11	0.10	−0.01	0.11	0.94
Missing interaction	1.00	0.11	0.11	0.00	0.11	0.94
Missing outcome (Y)−n=1000						
Correct imputation model	0.99	0.08	0.08	−0.01	0.08	0.96
Oversaturated imputation model^a^	1.00	0.08	0.08	0.00	0.08	0.96
Complete case analysis	0.99	0.07	0.07	−0.01	0.07	0.95
Missing interaction	1.00	0.07	0.07	0.00	0.07	0.96
Missing outcome (Y)−n=2000						
Correct imputation model	1.00	0.05	0.05	0.00	0.05	0.95
Oversaturated imputation model^a^	1.00	0.05	0.05	0.00	0.05	0.94
Complete case analysis	1.00	0.05	0.05	0.00	0.05	0.95
Missing interaction	1.00	0.05	0.05	0.00	0.05	0.95

*Abbreviations: n* = Sample size; Est. = Estimate; MC SE = Monte Carlo Standard Error; Avg. SE = Average Estimated Standard Error; 95% Cov. = 95% Confidence Interval Coverage.

^a^ Oversaturated imputation models fit split by exposure, include the outcome, and include the precision and confounder fit with natural splines with three degrees of freedom each.

**TABLE 3:** Simulation results when the data were generated with a nonlinear relationship between $Z_{P}$ and Y by imputation model specification. The true average treatment effect is 1.

Model specification	Est.	MC SE	Avg. SE	Bias	RMSE	95% Cov.
Missing confounder $(Z_{C})-n=100$						
Correct nonlinear imputation model	1.02	0.45	0.39	0.02	0.45	0.91
Misspecified precision variable	0.85	0.54	0.49	−0.15	0.56	0.91
Misspecified precision variable, Missing interaction	0.92	0.46	0.46	−0.08	0.46	0.94
Precision Variable Linear in both Imputatation and Analysis Model	1.03	0.71	0.60	0.03	0.71	0.92
Missing confounder $(Z_{C})-n=500$						
Correct nonlinear imputation model	0.98	0.19	0.17	−0.02	0.19	0.91
Misspecified precision variable	0.80	0.24	0.24	−0.20	0.32	0.88
Misspecified precision variable, Missing interaction	0.86	0.21	0.23	−0.14	0.25	0.93
Precision Variable Linear in both Imputatation and Analysis Model	0.99	0.29	0.29	−0.01	0.29	0.95
Missing confounder $(Z_{C})-n=1000$						
Correct nonlinear imputation model	1.01	0.13	0.12	0.01	0.13	0.95
Misspecified precision variable	0.81	0.17	0.18	−0.19	0.25	0.87
Misspecified precision variable, Missing interaction	0.85	0.16	0.17	−0.15	0.22	0.91
Precision Variable Linear in both Imputatation and Analysis Model	1.01	0.21	0.21	0.01	0.21	0.95
Missing confounder $(Z_{C})-n=2000$						
Correct nonlinear imputation model	1.00	0.09	0.09	0.00	0.09	0.94
Misspecified precision variable	0.81	0.12	0.13	−0.19	0.22	0.76
Misspecified precision variable, Missing interaction	0.85	0.12	0.13	−0.15	0.19	0.85
Precision Variable Linear in both Imputatation and Analysis Model	0.99	0.14	0.15	−0.01	0.14	0.95
Missing outcome (Y)−n=100						
Correct nonlinear imputation model	1.01	0.48	0.42	0.01	0.48	0.92
Misspecified precision variable	0.89	1.42	1.10	−0.11	1.42	0.93
Misspecified precision variable, Missing interaction	0.88	1.49	1.09	−0.12	1.49	0.94
Missing outcome (Y)−n=500						
Correct nonlinear imputation model	1.00	0.18	0.18	0.00	0.18	0.94
Misspecified precision variable	0.91	0.66	0.62	−0.09	0.67	0.95
Misspecified precision variable, Missing interaction	1.01	0.67	0.63	0.01	0.67	0.96
Missing outcome (Y)−n=1000						
Correct nonlinear imputation model	1.00	0.13	0.13	0.00	0.13	0.94
Misspecified precision variable	0.96	0.49	0.47	−0.04	0.49	0.95
Misspecified precision variable, Missing interaction	1.07	0.50	0.49	0.07	0.50	0.97
Missing outcome (Y)−n=2000						
Correct nonlinear imputation model	1.00	0.09	0.09	0.00	0.09	0.96
Misspecified precision variable	1.01	0.36	0.36	0.01	0.36	0.96
Misspecified precision variable, Missing interaction	1.05	0.33	0.35	0.05	0.33	0.97

*Abbreviations: n* = Sample size; Est. = Estimate; MC SE = Monte Carlo Standard Error; Avg. SE = Average Estimated Standard Error; 95% Cov. = 95% Confidence Interval Coverage.

The results reveal several important patterns in causal effect estimation with missing data. Correctly specified imputation models consistently yielded unbiased estimates of the average treatment effect across all missingness scenarios, with mean estimates close to the true value of Δ=1 (Tables 1, 2, 3). Likewise, the oversaturated imputation model, despite containing additional nonlinear terms, was also unbiased and had proper coverage. Complete case analysis was unbiased when $Z_{C}$ was missing because the outcome model was correctly specified and included X, which was the factor that dictated whether $Z_{C}$ was missing. However, the complete case analysis was biased when Y was missing despite the fact that the missingness was at random (based on $Z_{C}$). This underscores the importance of understanding the relationship between the missing data mechanism and the causal structure when deciding whether complete case analysis is appropriate.

When $Z_{C}$ (confounder) was missing, omitting $Z_{P}$ from the imputation model resulted in substantial bias in the estimated effect between X and Y, despite $Z_{P}$ being a precision variable that affects only the outcome. Omitting Y or X when $Z_{C}$ was missing likewise produced a biased estimate (Table 1, Figure 4).

When Y (outcome) was missing, the patterns differed. Omitting $Z_{C}$ or $Z_{P}$ had minimal impact on bias (as would be expected from Section 3.2), but omitting X biased estimates downward. While omitting $Z_{P}$ did not have an impact on the bias with respect to the relationship between X and Y, it did have a large impact on the precision, as indicated by a higher average standard deviation (Table 1).

Since we used a doubly robust estimator that effectively included an interaction in the outcome model, for the imputation model to be congenial, we needed this interaction in the imputation model too. One approach to handle this is to separately fit the imputation model among the exposed and unexposed groups, which is what we did here. This was necessary for estimating the causal effect when Y was missing, but not for the scenarios we examined when $Z_{C}$ was missing. This separate modeling approach was also not required when the true data‐generating mechanism did not have treatment effect heterogeneity (Table 2).

In nonlinear scenarios, misspecifying the functional form of variables produced a similar bias to omitting them entirely. For instance, using the wrong form for $Z_{P}$ when $Z_{C}$ is missing yielded bias of the same magnitude as completely omitting $Z_{P}$ (Table 3). We also examined a scenario where both the nonlinear term was misspecified as linear and the interaction between X and $Z_{C}$ was not properly accounted for in the imputation model. Because omitting the nonlinearity biases results downward while omitting the interaction biases results upward, these opposing biases partially offset each other, resulting in estimates that appear slightly better than those from misspecifying nonlinearity alone (Table 3). This phenomenon illustrates an important methodological point that apparently “good” performance from a misspecified model may actually reflect the fortuitous cancellation of multiple sources of bias rather than correct model specification. Such scenarios have led to misinterpretation of results in previous studies [11], where researchers may incorrectly conclude that particular methods are adequate when the observed performance actually stems from compensating errors.

Examining scenarios with multiple confounders where one was missing and the other had various nonlinear relationships with the exposure and outcome (Tables 4, 5, 6), we find that imputation model sensitivity depends on whether the nonlinear relationship involves the exposure or the outcome. When the nonlinear relationship is between the additional confounder ($Z_{C2}$) and the exposure, the imputation model is not sensitive to this misspecification (assuming separate imputation models are correctly specified by exposure status) (Table 4). However, when the nonlinear relationship is between the additional confounder and the outcome, incorrectly specifying $Z_{C2}$ in the imputation model as linear results in substantial bias (Tables 5 and 6). Across all scenarios, the oversaturated imputation model recovers unbiased estimates, provided the sample size is sufficient (i.e., it performs poorly when n=100).

**TABLE 4:** Simulation results when the data were generated with a nonlinear relationship between $Z_{C2}$ and X by imputation model specification. The true average treatment effect is 1.

Model specification	Est.	MC SE	Avg. SE	Bias	RMSE	95% Cov.
Missing confounder $(Z_{C1})-n=100$						
Correct imputation model	0.97	0.40	0.33	−0.03	0.40	0.87
Oversaturated imputation model^a^ ^a^	0.92	0.44	0.34	−0.08	0.45	0.82
Misspecified confounder $\left(Z_{C2}\right)$	1.01	0.40	0.31	0.01	0.40	0.85
Missing confounder $(Z_{C1})-n=500$						
Correct imputation model	0.97	0.21	0.18	−0.03	0.21	0.87
Oversaturated imputation model^a^	0.98	0.21	0.17	−0.02	0.21	0.86
Misspecified confounder $\left(Z_{C2}\right)$	0.99	0.21	0.17	−0.01	0.21	0.87
Missing confounder $(Z_{C1})-n=1000$						
Correct imputation model	0.98	0.16	0.14	−0.02	0.16	0.88
Oversaturated imputation model^a^	1.01	0.17	0.14	0.01	0.17	0.88
Misspecified confounder ($Z_{C2}$)	0.99	0.16	0.14	−0.01	0.16	0.90
Missing confounder $(Z_{C1})-n=2000$						
Correct imputation model	0.97	0.14	0.10	−0.03	0.14	0.85
Oversaturated imputation model^a^	1.00	0.14	0.11	0.00	0.14	0.85
Misspecified confounder ($Z_{C2}$)	0.99	0.14	0.11	−0.01	0.14	0.88
Missing outcome (Y)−n=100						
Correct imputation model	1.00	0.54	0.40	0.00	0.54	0.86
Oversaturated imputation model^a^	0.97	0.71	0.58	−0.03	0.71	0.88
Misspecified Confounder ($Z_{C2}$)	1.02	0.45	0.34	0.02	0.45	0.85
Missing outcome (Y)−n=500						
Correct imputation model	1.00	0.23	0.20	0.00	0.23	0.89
Oversaturated imputation model^a^	1.01	0.23	0.19	0.01	0.23	0.87
Misspecified confounder ($Z_{C2}$)	1.01	0.21	0.18	0.01	0.21	0.89
Missing outcome (Y)−n=1000						
Correct imputation model	0.98	0.19	0.16	−0.02	0.19	0.89
Oversaturated imputation model^a^	1.00	0.17	0.15	0.00	0.17	0.90
Misspecified confounder $\left(Z_{C2}\right)$	0.99	0.16	0.14	−0.01	0.16	0.89
Missing outcome (Y)−n=2000						
Correct imputation model	1.00	0.14	0.12	0.00	0.14	0.91
Oversaturated imputation model^a^	1.00	0.14	0.11	0.00	0.14	0.89
Misspecified confounder $\left(Z_{C2}\right)$	1.00	0.12	0.11	0.00	0.12	0.90

*Abbreviations: n* = Sample size; Est. = Estimate; MC SE = Monte Carlo Standard Error; Avg. SE = Average Estimated Standard Error; 95% Cov. = 95% Confidence Interval Coverage.

^a^ Oversaturated imputation models fit split by exposure, include the outcome, and include the precision and confounder fit with natural splines with three degrees of freedom each.

**TABLE 5:** Simulation results when the data were generated with a nonlinear relationship between $Z_{C2}$ and Y by imputation model specification. The true average treatment effect is 1.

Model specification	Est.	MC SE	Avg. SE	Bias	RMSE	95% Cov.
Missing confounder $(Z_{C1})-n=100$						
Correct imputation model	0.96	1.73	1.21	−0.04	1.73	0.82
Oversaturated imputation model*	0.77	1.96	1.29	−0.23	1.97	0.80
Misspecified confounder ($Z_{C2}$)	0.47	1.91	1.36	−0.53	1.98	0.82
Missing confounder $(Z_{C1})-n=500$						
Correct imputation model	0.97	0.72	0.56	−0.03	0.72	0.84
Oversaturated imputation model^a^	0.96	0.72	0.60	−0.04	0.72	0.86
Misspecified confounder ($Z_{C2}$)	0.41	0.93	0.78	−0.59	1.10	0.82
Missing confounder $(Z_{C1})-n=1000$						
Correct imputation model	1.04	0.52	0.44	0.04	0.52	0.88
Oversaturated imputation model^a^	0.97	0.57	0.46	−0.03	0.57	0.88
Misspecified confounder ($Z_{C2}$)	0.37	0.84	0.69	−0.63	1.05	0.84
Missing confounder $(Z_{C1})-n=2000$						
Correct imputation model	1.02	0.37	0.32	0.02	0.37	0.90
Oversaturated imputation model^a^	0.98	0.40	0.35	−0.02	0.40	0.89
Misspecified confounder ($Z_{C2}$)	0.32	0.67	0.60	−0.68	0.95	0.85
Missing outcome (Y)−n=100						
Correct imputation model	1.10	2.02	1.36	0.10	2.02	0.81
Oversaturated imputation model^a^	1.02	2.86	1.90	0.02	2.86	0.84
Misspecified confounder ($Z_{C2}$)	−0.15	2.59	1.71	−1.15	2.83	0.79
Missing outcome (Y)−n=500						
Correct imputation model	1.04	0.77	0.60	0.04	0.77	0.85
Oversaturated imputation model^a^	0.86	0.85	0.64	−0.14	0.86	0.84
Misspecified confounder ($Z_{C2}$)	0.02	1.45	1.00	−0.98	1.75	0.82
Missing outcome (Y)−n=1000						
Correct imputation model	1.02	0.52	0.44	0.02	0.52	0.89
Oversaturated imputation model^a^	0.94	0.58	0.47	−0.06	0.59	0.88
Misspecified confounder ($Z_{C2}$)	0.05	1.12	0.82	−0.95	1.47	0.77
Missing outcome (Y)−n=2000						
Correct imputation model	0.98	0.40	0.34	−0.02	0.40	0.89
Oversaturated imputation model^a^	0.95	0.44	0.36	−0.05	0.44	0.89
Misspecified confounder ($Z_{C2}$)	−0.03	0.97	0.69	−1.03	1.42	0.69

*Abbreviations: n* = Sample size; Est. = Estimate; MC SE = Monte Carlo Standard Error; Avg. SE = Average Estimated Standard Error; 95% Cov. = 95% Confidence Interval Coverage.

^a^ Oversaturated imputation models fit split by exposure, include the outcome, and include the precision and confounder fit with natural splines with three degrees of freedom each.

**TABLE 6:** Simulation results when the data were generated with a nonlinear relationship between both $Z_{C2}$ and X and $Z_{C2}$ and Y by imputation model specification. The true average treatment effect is 1.

Model specification	Est.	MC SE	Avg. SE	Bias	RMSE	95% Cov.
Missing confounder $(Z_{C1})-n=100$						
Correct imputation model	1.13	3.78	2.43	0.13	3.78	0.75
Oversaturated imputation model^a^	0.66	4.07	2.61	−0.34	4.08	0.74
Misspecified confounder ($Z_{C2}$)	0.31	3.65	2.54	−0.69	3.71	0.76
Missing confounder $(Z_{C1})-n=500$						
Correct imputation model	1.12	1.65	1.16	0.12	1.66	0.78
Oversaturated imputation model^a^	0.83	1.77	1.21	−0.17	1.78	0.77
Misspecified confounder ($Z_{C2}$)	0.31	1.82	1.43	−0.69	1.95	0.80
Missing confounder $(Z_{C1})-n=1000$						
Correct imputation model	1.02	1.36	0.95	0.02	1.36	0.78
Oversaturated imputation model^a^	1.02	1.37	0.99	0.02	1.36	0.80
Misspecified confounder ($Z_{C2}$)	0.11	1.58	1.25	−0.89	1.81	0.79
Missing confounder $(Z_{C1})-n=2000$						
Correct imputation model	1.06	1.10	0.73	0.06	1.10	0.80
Oversaturated imputation model^a^	1.00	1.06	0.78	0.00	1.06	0.80
Misspecified confounder ($Z_{C2}$)	0.15	1.40	1.03	−0.85	1.64	0.80
Missing outcome (Y)−n=100						
Correct imputation model	1.34	4.79	2.98	0.34	4.80	0.76
Oversaturated imputation model^a^	0.43	8.14	5.62	−0.57	8.15	0.81
Misspecified confounder ($Z_{C2}$)	−0.01	4.44	2.96	−1.01	4.55	0.77
Missing outcome (Y)−n=500						
Correct imputation model	1.10	1.79	1.36	0.10	1.79	0.79
Oversaturated imputation model^a^	1.03	1.91	1.43	0.03	1.91	0.79
Misspecified confounder ($Z_{C2}$)	−0.16	2.34	1.58	−1.16	2.61	0.78
Missing outcome (Y)−n=1000						
Correct imputation model	0.89	1.38	1.03	−0.11	1.38	0.80
Oversaturated imputation model^a^	0.91	1.37	1.07	−0.09	1.37	0.80
Misspecified confounder ($Z_{C2}$)	−0.23	2.06	1.26	−1.23	2.40	0.72
Missing outcome (Y)−n=2000						
Correct imputation model	1.02	1.01	0.79	0.02	1.01	0.83
Oversaturated imputation model^a^	0.85	1.12	0.82	−0.15	1.13	0.81
Misspecified confounder ($Z_{C2}$)	0.06	1.67	1.04	−0.94	1.92	0.76

*Abbreviations: n* = Sample size; Est. = Estimate; MC SE = Monte Carlo Standard Error; Avg. SE = Average Estimated Standard Error; 95% Cov. = 95% Confidence Interval Coverage.

^a^ Oversaturated imputation models fit split by exposure, include the outcome, and include the precision and confounder fit with natural splines with three degrees of freedom each.

Overall, these findings confirm that a proper imputation model specification requires including all variables that appear in either the propensity score model or outcome model, in their correct functional form. The bias patterns demonstrate that omitting seemingly secondary variables like precision variables ($Z_{P}$) can substantially compromise causal effect estimation, as well as highlight the importance of considering the functional form (e.g., the presence or interactions of an nonlinear terms) in the subsequent models.

## 6 Implementation

To illustrate the recommended workflow, here we walk through the nonlinear case with treatment heterogeneity used in the simulation study (Figure 2). Below we (i) generate one dataset under the scenario described in Section 4.1, (ii) induce missingness in the confounder, (iii) impute the confounder separately by exposure to preserve congeniality with an outcome model that includes an interaction, (iv) estimate the causal effect in each imputed dataset using IPW and a nonlinear outcome model, and (v) pool estimates using Rubin's rules. We set m=20 imputations throughout.

For the analysis we created a helper fit_ipw_effect() which fits propensity score weights (using weightit from the WeightIt package [29]), fits the weighted outcome model (using lm_weightit from the WeightIt package), and extracts the average treatment effect via avg_comparisons() from the marginaleffects package [30].

LISTING

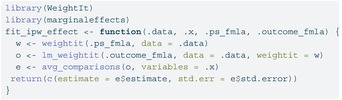

### 6.1 Data Generation

We simulate the same data‐generating mechanism used in the Section 4.1: Three continuous covariates $Z_{I}$ (zi), $Z_{P}$ (zp) and $Z_{C}$ (zc), a binary exposure X and an outcome that is nonlinear in $Z_{P}$ and includes an $X\times Z_{C}$ interaction. Missingness is induced in $Z_{C}$ with probability depending on X to achieve approximately 20% missingness.

LISTING

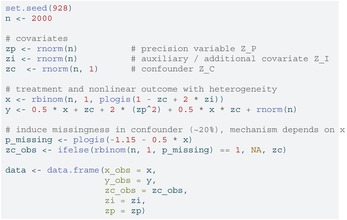

### 6.2 Multiple Imputation (Separate by Exposure)

To ensure congeniality with the outcome model that includes an $X\times Z_{C}$ interaction, we impute zc_obs separately within the exposed and unexposed strata. For simplicity, we load the tidyverse package [31] for data manipulation (filtering to split the data by the exposure) and recombining the completed datasets (with map). The imputation model includes the outcome y_obs and a spline term for the precision variable zp to capture nonlinearity. The make.formulas function from the mice package is a helper function that creates formulas for each of the variables in a data frame. By default, it includes all other variables present in the data frame in an additive model (with no nonlinear terms or interactions). We can update components of this object as needed, for example, here we will update to include the nonlinear term for zp. We then use the mice function to perform the imputations and complete to create the m=20 complete datasets.

LISTING

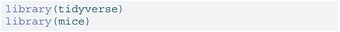

LISTING

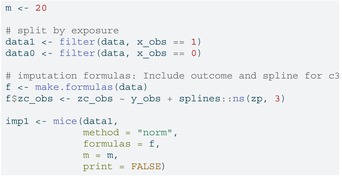

LISTING

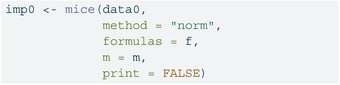

LISTING

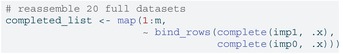

### 6.3 Analysis Within Each Imputed Dataset

For each completed dataset, we estimate propensity score weights with x_obs – zc_obs and then fit the weighted nonlinear outcome regression that includes a cubic spline for zp, and the interaction $x_{-}\mathrm{obs}\;:\;zc_{-}\mathrm{obs}$. The ATE is extracted via the $\mathrm{fi}t_{-}\mathrm{ip}w_{-}\mathrm{effect}()$ helper function that we created above. This will output an estimate and standard error for each of the 20 imputed datasets.

LISTING

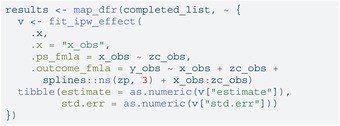

### 6.4 Pooling (Rubin's Rules)

We combine the point estimates ${\hat{\Delta }}^{\left(j\right)}$ and their within‐imputation variances $U^{\left(j\right)}$ across the m completed datasets using Rubin's rules. The pooled estimate is $\bar{\Delta }=m^{-1}\sum_{j}{\hat{\Delta }}^{\left(j\right)}$ and the total variance is $T=Ū+(1+m^{-1})B$ (as described in Section 2).

LISTING

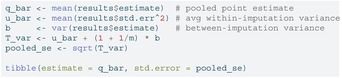

LISTING

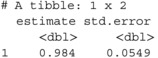

We can now report the estimate and std.errorabove as the pooled ATE and its standard error.

## 7 Conclusion

This study provides clear guidance on the proper specification of imputation models when combining multiple imputation with propensity score methods for causal inference. A central takeaway is the critical importance of representing variables in their correct functional form within the imputation model, a detail often overlooked in practice. While commonly used software, such as mice [28], include all variables by default (a good practice), the default is to include them linearly, which can introduce bias when the relationships in analysis models are treated as nonlinear.

Our findings demonstrate that the imputation model must include all variables used in either the propensity score model or the outcome model, not just in name, but in the same functional form as in the analysis models. This ensures congeniality and supports valid estimation of treatment effects. The separate patterns observed when either confounders ($Z_{C}$) or outcomes (Y) are missing highlight the need for careful consideration based on the specific missing data scenario.

An important challenge for practitioners is that there may not be a shared understanding of what is meant by including “all” variables in the imputation model. For some researchers, “all” may refer only to confounders, excluding the exposure or outcome variables. Others may interpret it as including every variable in the dataset but only in linear form, regardless of how they are specified in the analysis models. Still others may consider only main effects as sufficient, overlooking interaction terms or nonlinear transformations that appear in their analysis models. This lack of consensus around what constitutes a complete imputation model specification can lead to seemingly well‐intentioned but ultimately inadequate implementations. The issue is compounded by the fact that many practitioners may believe they are following best practices by including “all variables” without recognizing that their interpretation of completeness differs from what congeniality actually requires. Establishing clearer operational definitions of comprehensive imputation model specification, one that explicitly encompasses exposure variables, outcome variables, appropriate functional forms, and relevant interactions, is essential for moving the field toward more consistent and valid practice.

While the principle of congeniality is well established within the missing data methodological community, its importance may not always be fully recognized or consistently applied across different areas of research, particularly in scenarios where multiple analysis models are used. These principles of careful imputation model specification apply broadly across different types of analyses involving multiple models, not only in causal inference settings. Whether the research goal is prediction, description, or causal explanation, ensuring that imputation models adequately reflect the complexity and functional forms used in subsequent analyses remain critical for valid statistical inference. Our theoretical and simulated results show that excluding variables from the imputation model, even those that are not confounders (e.g., precision variables) can introduce substantial bias in the treatment effect estimates when confounders are imputed. This bias remains even when using doubly robust estimators (and potentially worsens, as this introduces an additional model that the imputation model must be congenial with).

In scenarios with nonlinear relationships, we found that misspecifying the functional form of a variable in the imputation model led to bias similar in magnitude to excluding the variable entirely. This result underscores the need for careful alignment between the imputation model and the analysis models. Our findings regarding the treatment effect heterogeneity further emphasizes this point, as we demonstrated that separate imputation models for exposed and unexposed groups may be necessary when the outcome is missing and the true data‐generating process includes treatment effect heterogeneity.

The complete case analysis results offer additional nuance to our understanding, showing that complete case analysis can be appropriate in some circumstances (when confounders are missing under specific conditions) but biased in others (when the outcome is missing), even with missing at random mechanisms. This highlights the complex interplay between causal structure and missing data mechanisms.

While this work focused on continuous outcomes with a binary treatment, similar principles apply to other outcome types and data structures. Time‐to‐event outcomes present additional complexities for multiple imputation, as the imputation model must appropriately handle censoring mechanisms and potentially time‐varying effects. The bias from imputation model misspecification may be even more pronounced in survival settings due to the challenges of adequately representing the hazard function and its relationship with covariates in the imputation model.

The key practical recommendations from our study are:

1. Include all variables from the propensity score or the outcome models in the imputation model, regardless of their causal role (confounder and precision variables), along with the exposure (when imputing a confounder or the outcome), and the outcome (when imputing a confounder).
2. Ensure that these variables are included in the same functional form as they appear in the analysis models (e.g., if quadratic terms are used in the analysis, they should also be included in the imputation).
3. When treatment effect heterogeneity is accounted for in the analysis modeling procedure, fit separate imputation models by treatment status.
4. Understand the relationship between missing data mechanisms and causal structure when deciding whether complete case analysis is appropriate.
5. When in doubt, err on the side of inclusion rather than exclusion when specifying the imputation model.

These recommendations align with fundamental principles of proper multiple imputation. The imputation model must be at least as rich as the analysis model to preserve the joint distribution of all variables relevant to the analysis. Our study extends these principles to the specific context of causal inference, where multiple models (propensity score and outcome) may be involved. By following these guidelines, researchers can more confidently apply multiple imputation in observational studies where both missing data and confounding are present, leading to more valid and reliable estimates of causal effects.

## Funding

The author has nothing to report.

## Conflicts of Interest

The author declares no conflicts of interest.

## References

[1] D. O. Scharfstein , A. Rotnitzky , and J. M. Robins , “Adjusting for Nonignorable Drop‐Out Using Semiparametric Nonresponse Models,” Journal of the American Statistical Association 94, no. 448 (1999): 1096–1120.

[2] J. M. Robins , “Robust Estimation in Sequentially Ignorable Missing Data and Causal Inference Models,” in Proceedings of the American Statistical Association Section on Bayesian Statistical Science (2000), 6–10.

[3] J. K. Lunceford and M. Davidian , “Stratification and Weighting via the Propensity Score in Estimation of Causal Treatment Effects: A Comparative Study,” Statistics in Medicine 23, no. 19 (2004): 2937–2960. 15351954 10.1002/sim.1903

[4] H. Bang and J. M. Robins , “Doubly Robust Estimation in Missing Data and Causal Inference Models,” Biometrics 61, no. 4 (2005): 962–973. 16401269 10.1111/j.1541-0420.2005.00377.x

[5] J. D. Kang and J. L. Schafer , “Demystifying Double Robustness: A Comparison of Alternative Strategies for Estimating a Population Mean From Incomplete Data,” Statistical Science 22, no. 4 (2007): 523–539. 10.1214/07-STS227 PMC2397555 18516239

[6] M. J. Van der Laan and S. Rose , Targeted Learning: Causal Inference for Observational and Experimental Data, vol. 4 (Springer, 2011).

[7] E. Granger , J. C. Sergeant , and M. Lunt , “Avoiding Pitfalls When Combining Multiple Imputation and Propensity Scores,” Statistics in Medicine 38, no. 26 (2019): 5120–5132. 31512265 10.1002/sim.8355 PMC6856837

[8] C. Leyrat , S. R. Seaman , I. R. White , et al., “Propensity Score Analysis With Partially Observed Covariates: How Should Multiple Imputation Be Used?,” Statistical Methods in Medical Research 28, no. 1 (2019): 3–19. 28573919 10.1177/0962280217713032 PMC6313366

[9] M. Daniels , C. Wang , and B. Marcus , “Fully Bayesian Inference Under Ignorable Missingness in the Presence of Auxiliary Covariates,” Biometrics 70, no. 1 (2014): 62–72. 24571539 10.1111/biom.12121 PMC4007313

[10] J. W. Bartlett and R. A. Hughes , “Bootstrap Inference for Multiple Imputation Under Uncongeniality and Misspecification,” Statistical Methods in Medical Research 29, no. 12 (2020): 3533–3546. 32605503 10.1177/0962280220932189 PMC7682506

[11] R. Mitra and J. P. Reiter , “A Comparison of Two Methods of Estimating Propensity Scores After Multiple Imputation,” Statistical Methods in Medical Research 25, no. 1 (2016): 188–204. 22687877 10.1177/0962280212445945

[12] B. de Penning Vries and R. H. Groenwold , “Comments on Propensity Score Matching Following Multiple Imputation,” (2016). 10.1177/0962280216674296 27852808

[13] L. D'Agostino McGowan , S. C. Lotspeich , and S. A. Hepler , “The Why Behind Including y in Your Imputation Model,” Statistical Methods in Medical Research 33, no. 6 (2024): 996–1020. 38625810 10.1177/09622802241244608

[14] E. J. Williamson , A. Forbes , and R. Wolfe , “Doubly Robust Estimators of Causal Exposure Effects With Missing Data in the Outcome, Exposure or a Confounder,” Statistics in Medicine 31, no. 30 (2012): 4382–4400. 23086504 10.1002/sim.5643

[15] S. G. Dashti , K. J. Lee , J. A. Simpson , I. R. White , J. B. Carlin , and M. Moreno‐Betancur , “Handling Missing Data When Estimating Causal Effects With Targeted Maximum Likelihood Estimation,” American Journal of Epidemiology 193, no. 7 (2024): 1019–1030. 38400653 10.1093/aje/kwae012 PMC11228874

[16] P. T. Von Hippel , “How to Impute Interactions, Squares, and Other Transformed Variables,” Sociological Methodology 39, no. 1 (2009): 265–291.

[17] I. R. White , P. Royston , and A. M. Wood , “Multiple Imputation Using Chained Equations: Issues and Guidance for Practice,” Statistics in Medicine 30, no. 4 (2011): 377–399. 21225900 10.1002/sim.4067

[18] P. T. von Hippel , “Should a Normal Imputation Model Be Modified to Impute Skewed Variables?,” Sociological Methods & Research 42, no. 1 (2013): 105–138.

[19] J. M. Robins , A. Rotnitzky , and L. P. Zhao , “Estimation of Regression Coefficients When Some Regressors Are Not Always Observed,” Journal of the American Statistical Association 89, no. 427 (1994): 846–866.

[20] J. Hahn , “Functional Restriction and Efficiency in Causal Inference,” Review of Economics and Statistics 86, no. 1 (2004): 73–76.

[21] M. A. Brookhart , S. Schneeweiss , K. J. Rothman , R. J. Glynn , J. Avorn , and T. Stürmer , “Variable Selection for Propensity Score Models,” American Journal of Epidemiology 163, no. 12 (2006): 1149–1156. 16624967 10.1093/aje/kwj149 PMC1513192

[22] X. De Luna , I. Waernbaum , and T. S. Richardson , “Covariate Selection for the Nonparametric Estimation of an Average Treatment Effect,” Biometrika 98, no. 4 (2011): 861–875.

[23] J. M. Franklin , W. Eddings , R. J. Glynn , and S. Schneeweiss , “Regularized Regression Versus the High‐Dimensional Propensity Score for Confounding Adjustment in Secondary Database Analyses,” American Journal of Epidemiology 182, no. 7 (2015): 651–659. 26233956 10.1093/aje/kwv108

[24] D. Tang , D. Kong , W. Pan , and L. Wang , “Ultra‐High Dimensional Variable Selection for Doubly Robust Causal Inference,” Biometrics 79, no. 2 (2023): 903–914. 35043393 10.1111/biom.13625

[25] J. A. Craycroft , J. Huang , and M. Kong , “Propensity Score Specification for Optimal Estimation of Average Treatment Effect With Binary Response,” Statistical Methods in Medical Research 29, no. 12 (2020): 3623–3640. 32640934 10.1177/0962280220934847

[26] S. Van Buuren and S. Van Buuren , Flexible Imputation of Missing Data, vol. 10 (CRC press, 2012).

[27] D. Rubin , Multiple Imputation for Nonresponse in Surveys (J. Wiley & Sons, 1987).

[28] S. van Buuren and K. Groothuis‐Oudshoorn , “Mice: Multivariate Imputation by Chained Equations in R,” Journal of Statistical Software 45, no. 3 (2011): 1–67, 10.18637/jss.v045.i03.

[29] N. Greifer , “WeightIt: Weighting for Covariate Balance in Observational Studies, R Package Version 1.4.0,” (2025), https://CRAN.R‐project.org/package=WeightIt.

[30] V. Arel‐Bundock , N. Greifer , and A. Heiss , “How to Interpret Statistical Models Using Marginaleffects for R and Python,” Journal of Statistical Software 111, no. 9 (2024): 1–32, 10.18637/jss.v111.i09.

[31] H. Wickham , M. Averick , J. Bryan , et al., “Welcome to the Tidyverse,” Journal of Open Source Software 4, no. 43 (2019): 1686, 10.21105/joss.01686.

